# Supplementary material for: Not getting in too deep: A practical deep learning approach to routine crystallisation image classification
Source: PLoS One. 2023 Mar 9;18(3):e0282562. doi: 10.1371/journal.pone.0282562 (PMC9997964; doi:10.1371/journal.pone.0282562)
Supplement: S1 File — (PDF) [file pone.0282562.s001.pdf]

Supplementary Information

Initial Learning Rate	$2e^{-4}$
Learning Rate Factor	0.5
Batch Size	16
Optimizer	Adam optimiser
Loss Function	Cross-Entropy
Epochs	100
Horizontal/Vertical flipping	Yes
Zoom Range	30%
Rotation Range	30°
Width/Height Shifting	5%
Re-scaling	1/255

Table S1: Optimal training and image augmentation parameters used to train classifiers with each chosen network architecture.

Cross-entropy loss function

For n classes, the cross-entropy loss function is defined as

$$L = -\sum_{i=1}^{n} t_i log(p_i$$

where t_i is the true probability for the ith class (1 for the correct class, 0 for other classes) and p_i is the Softmax probability for the ith class.

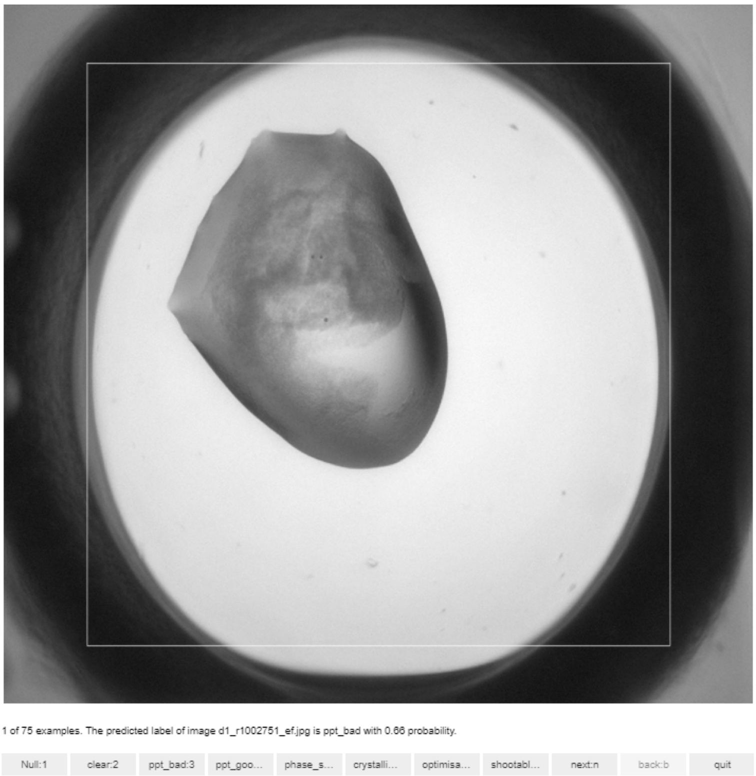

Figure S1: Screenshot showing the GUI used to check the results of labelling.

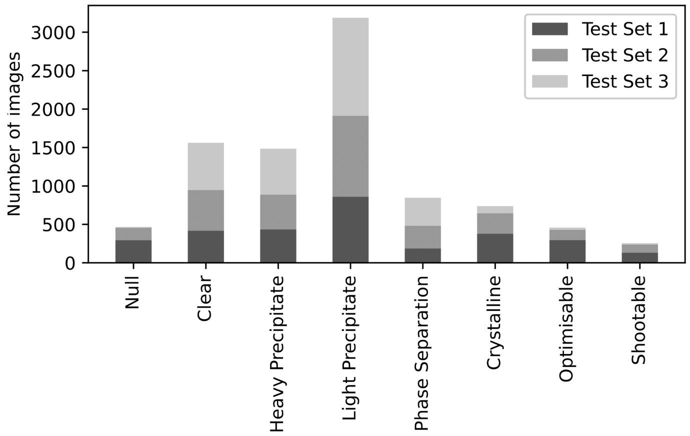

Figure S2: The number of images in each class for the three test data sets.

Test Sets	Test 1				Test 2				Test 3			
Classes	DenseNet121	ResNet50	InceptionV3	Xception	DenseNet121	ResNet50	InceptionV3	Xception	DenseNet121	ResNet50	InceptionV3	Xception
Top-1 Accuracy (%)	87.8	75.4	82.3	84.7	80.1	72.1	72.9	76.5	87.2	77.7	79.3	76.5
Adjacent Accuracy (%)	93.3	89.3	94.5	95.1	85.2	84.2	85.9	87.6	92.4	86.3	93.7	95.3
Kappa	0.86	0.71	0.79	0.82	0.75	0.66	0.67	0.71	0.83	0.69	0.73	0.77
F1	0.89	0.74	0.83	0.85	0.81	0.71	0.73	0.77	0.88	0.79	0.79	0.83
Precision	0.89	0.81	0.86	0.86	0.85	0.78	0.79	0.81	0.89	0.84	0.85	0.86

Table S2: The test results of all classes for each architecture and test set.

Network Architectures

Figures S3, S4 and S5 show the standard network architectures for DenseNet121, ResNet50 and Xception respectively while figures S6 and S7 show the standard network architecture for InceptionV3 (in two parts). The DenseNet121 architecture diagram is adapted from [1] with the other architecture diagrams adapted from [2]. All standard architectures were modified in the classification block in our study by adding a drop out layer and an additional fully connected layer as shown in Fig. S8.

References

[1] Chen S, Stromer D, Alabdalrahim HA, Schwab S, Weih M, Maier A. Automatic dementia screening and scoring by applying deep learning on clock-drawing tests. Scientific Reports. 2020;10(1):1–11.

[2] Leonardo MM, Carvalho TJ, Rezende E, Zucchi R, Faria FA. Deep Feature-Based Classifiers for Fruit Fly Identification (Diptera: Tephritidae). In: 2018 31st SIBGRAPI Conference on Graphics, Patterns and Images (SIBGRAPI); 2018. p. 41–47.

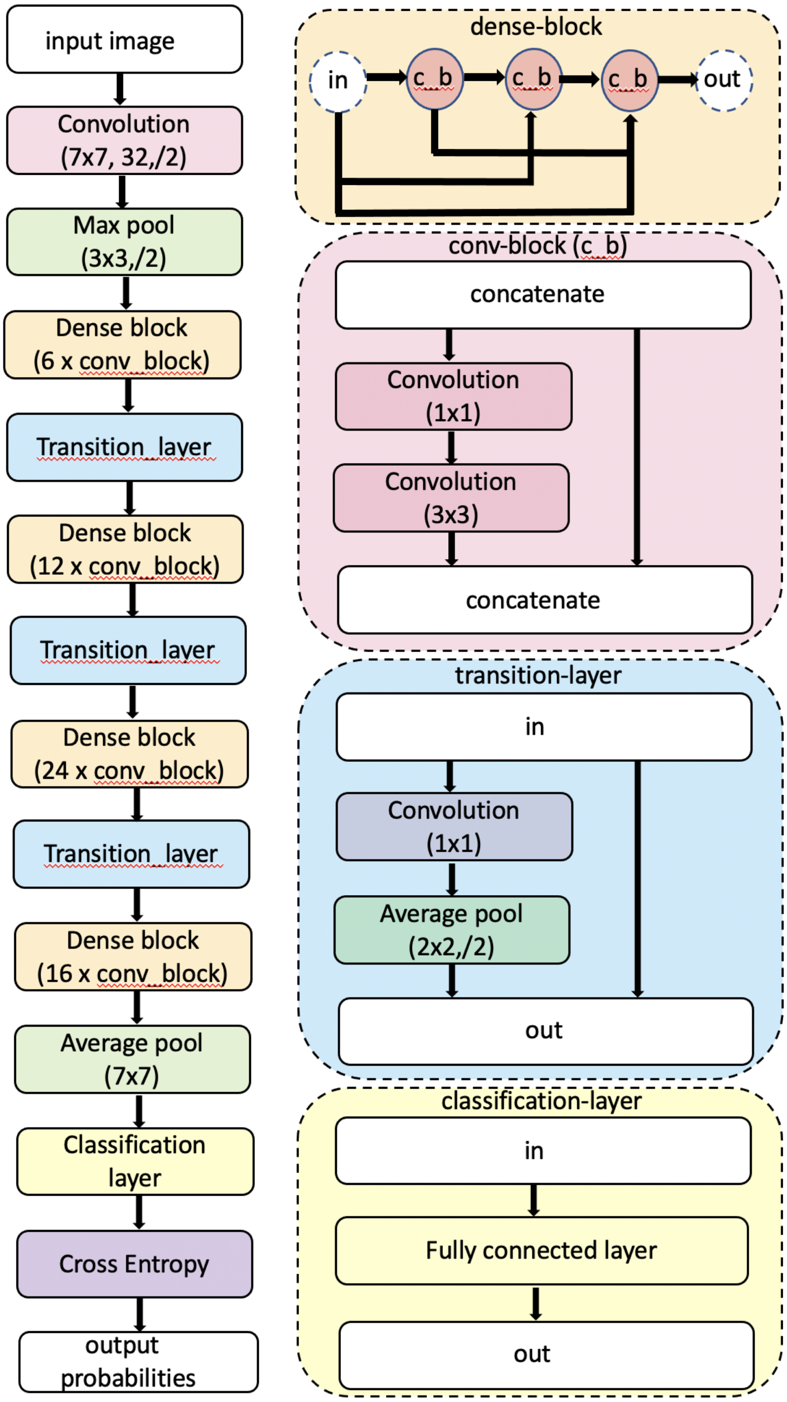

Figure S3: DenseNet121 network architecture.

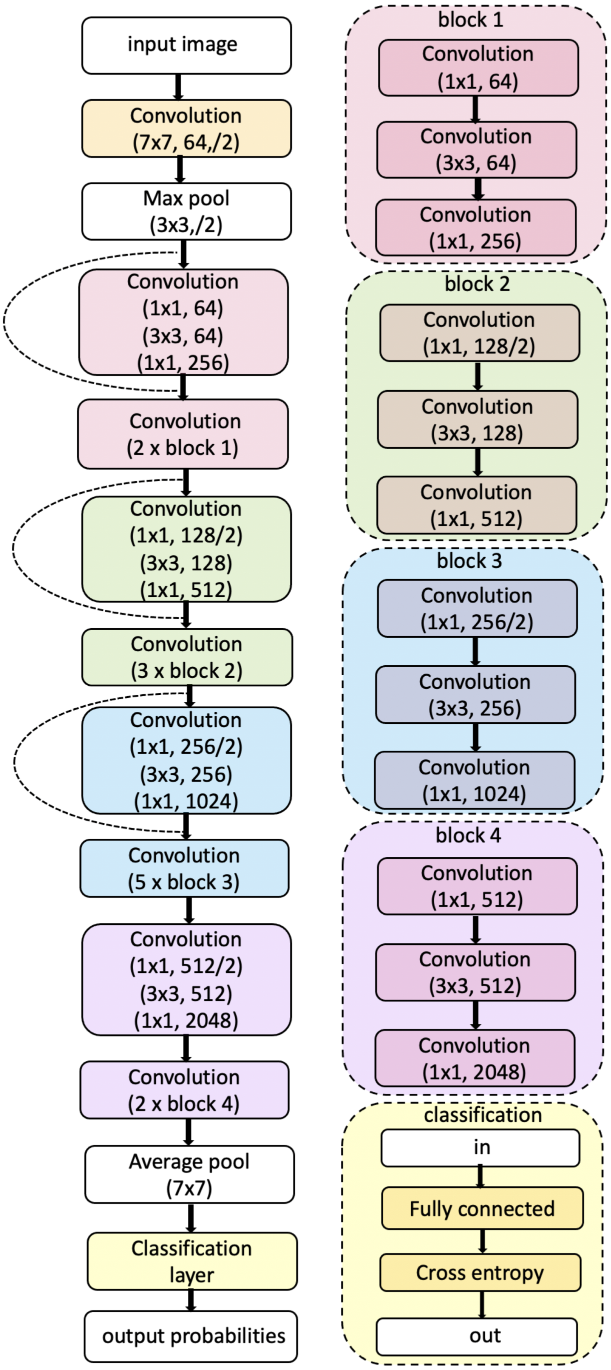

Figure S4: ResNet50 network architecture.

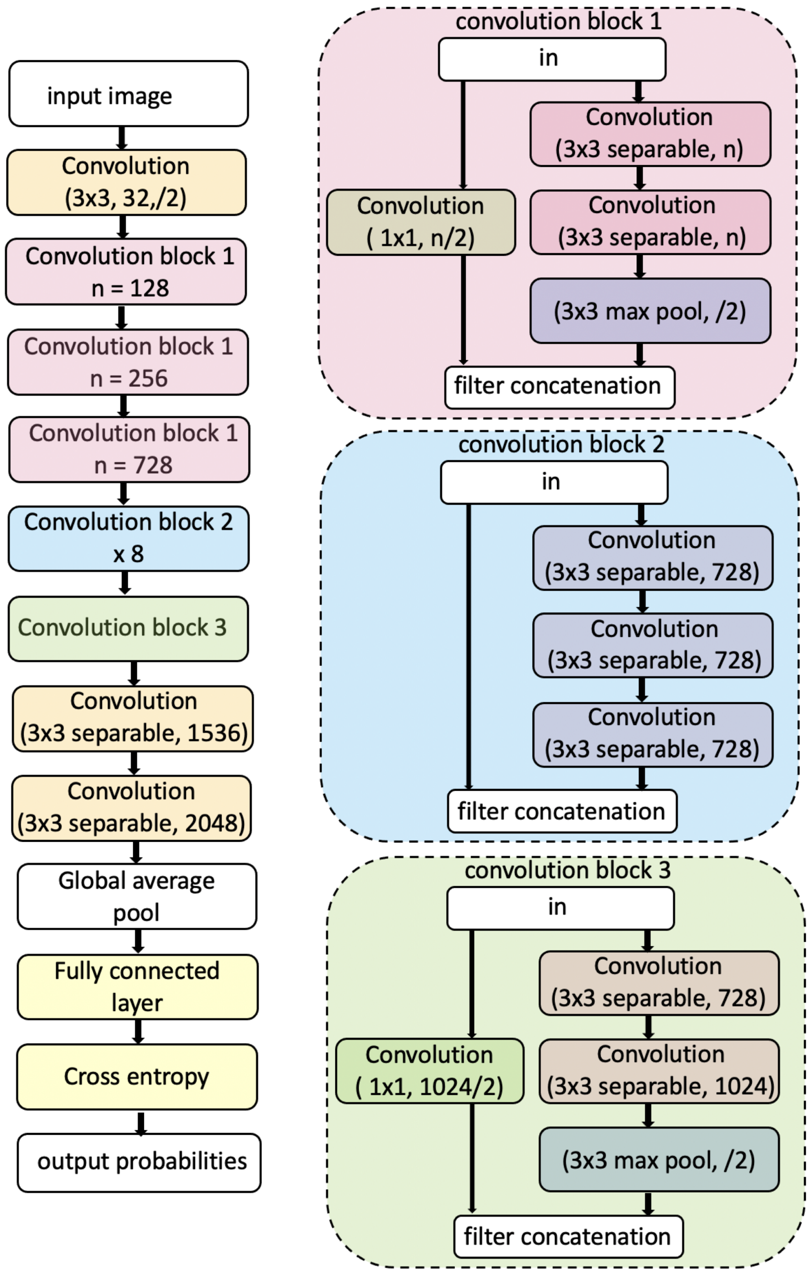

Figure S5: Xception network architecture.

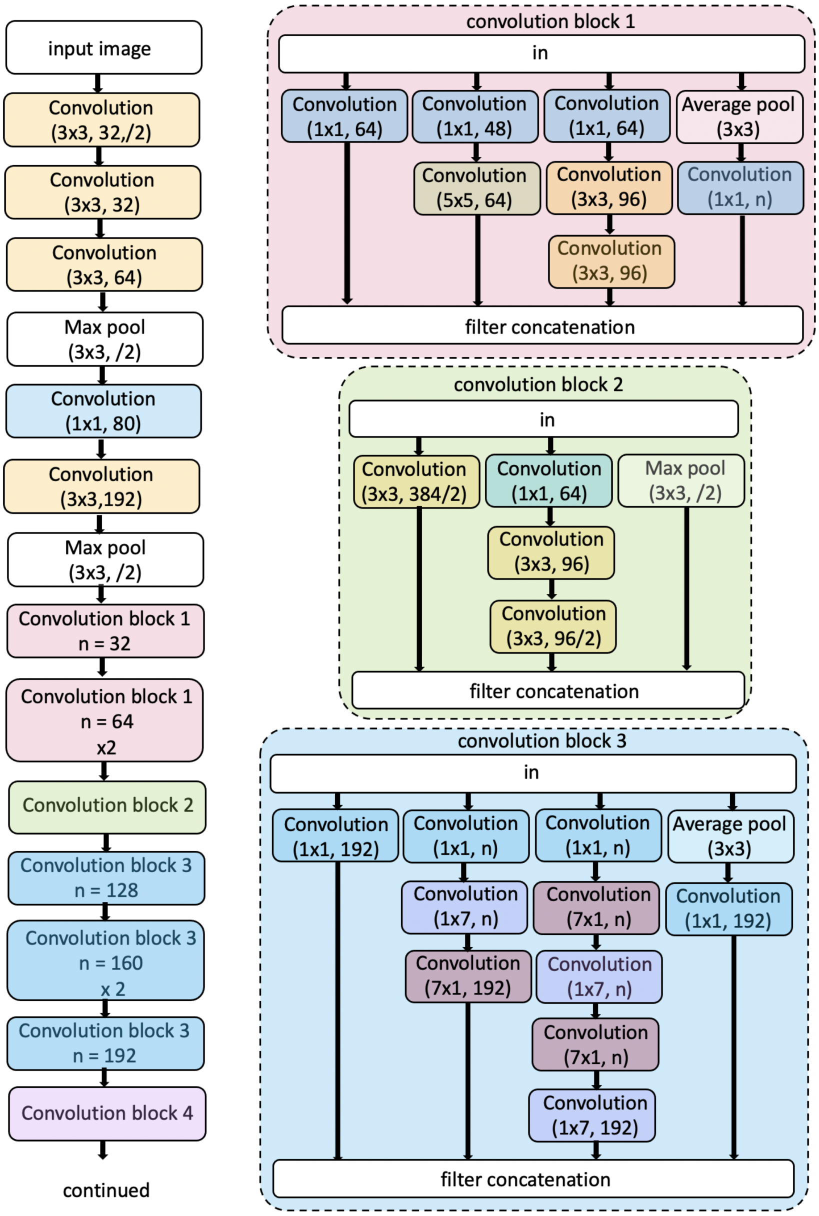

Figure S6: Inception network architecture.

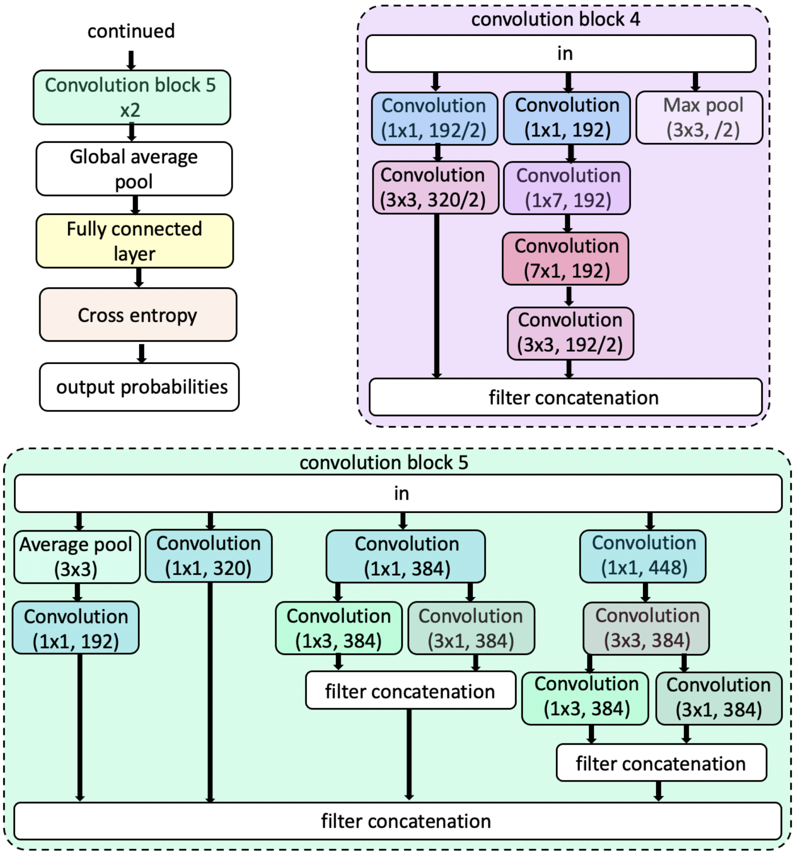

Figure S7: Inception network architecture continued.

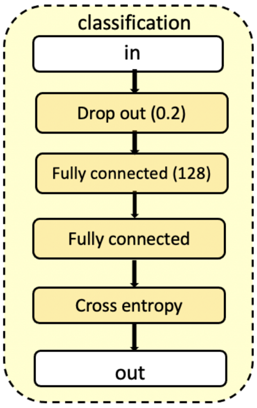

Figure S8: The modified classification block used in all our models.

Test set 1	null	clear	heavy-ppt	light-ppt	phase-sep	crystalline	optimisable	shootable
null	77.6	10.2	1.7	2.7	3.1	2.4	1.7	0.7
clear	0.0	96.2	0.0	1.0	0.5	1.9	0.5	0.0
heavy-ppt	0.5	0.9	74.9	13.1	2.8	7.6	0.2	0.0
light-ppt	0.2	0.8	1.9	90.6	3.4	2.9	0.2	0.0
phase-sep	0.0	0.0	0.0	0.0	95.2	4.3	0.5	0.0
crystalline	0.0	0.0	0.3	0.3	2.4	95.3	1.6	0.3
optimisable	0.3	0.0	0.0	0.0	2.4	15.2	79.4	2.7
shootable	0.0	0.0	0.0	0.0	0.0	2.3	1.5	96.2

Test set 2	null	clear	heavy-ppt	light-ppt	phase-sep	crystalline	optimisable	shootable
null	71.4	13.0	0.6	4.3	2.4	6.3	1.2	0.6
clear	0.2	89.8	0.2	0.2	0.6	8.1	0.9	0.0
heavy-ppt	1.1	0.2	58.2	13.5	2.1	23.5	0.9	0.4
light-ppt	0.0	0.9	1.0	82.1	6.3	8.4	1.1	0.1
phase-sep	0.0	0.0	1.7	0.0	93.2	4.1	0.0	0.0
crystalline	0.0	0.4	0.4	1.5	6.0	89.1	1.1	1.5
optimisable	0.0	0.0	5.9	4.4	8.1	20.7	52.6	8.1
shootable	0.0	0.0	0.9	0.0	1.9	5.6	0.9	90.7

Test set 3	null	clear	heavy-ppt	light-ppt	phase-sep	crystalline	optimisable	shootable
null	66.7	0.0	0.0	0.0	16.7	16.7	0.0	0.0
clear	0.0	94.1	0.0	0.2	0.6	1.5	4.1	0.0
heavy-ppt	0.2	0.0	74.7	17.7	2.8	3.8	0.7	0.0
light-ppt	0.0	0.2	2.1	86.6	7.8	3.1	0.2	0.0
phase-sep	0.0	0.0	0.0	0.5	98.9	0.5	0.0	0.0
crystalline	0.0	0.0	1.1	5.5	0.0	93.4	0.0	0.0
optimisable	0.0	7.4	0.0	29.6	0.0	7.4	55.5	0.0
shootable	0.0	0.0	0.0	0.0	0.0	0.0	0.0	100.0

Figure S9: Confusion matrix showing the results for each of the three test sets obtained using the DenseNet121 classifier. Rows show class labels with predicted class in columns.

Test set 1	null	clear	heavy-ppt	light-ppt	phase-sep	crystalline	optimisable	shootable
null	86.8	2.4	3.1	0.3	0.0	0.0	7.5	0.0
clear	13.9	85.2	0.0	0.2	0.0	0.0	0.7	0.0
heavy-ppt	0.7	3.0	67.4	26.4	1.1	0.5	0.9	0.0
light-ppt	2.8	3.5	2.4	87.7	2.2	0.2	1.2	0.0
phase-sep	1.1	3.2	0.0	2.1	86.1	0.5	7.0	0.0
crystalline	0.8	6.1	3.4	12.4	6.1	26.1	45.1	0.0
optimisable	0.0	0.3	0.0	0.0	0.0	0.7	99.0	0.0
shootable	0.8	0.8	0.0	2.3	0.0	0.0	58.0	38.2

Test set 2	null	clear	heavy-ppt	light-ppt	phase-sep	crystalline	optimisable	shootable
null	87.0	3.1	3.7	1.2	2.5	0.6	1.9	0.0
clear	2.8	95.5	0.0	0.2	0.8	0.0	0.8	0.0
heavy-ppt	1.8	4.4	66.6	12.2	4.9	1.5	8.6	0.0
light-ppt	2.5	4.1	6.5	76.8	8.2	0.2	1.8	0.0
phase-sep	0.7	2.0	0.7	2.0	75.3	0.3	19.0	0.0
crystalline	3.0	5.2	13.1	15.0	13.5	12.4	37.8	0.0
optimisable	0.0	0.0	0.0	2.2	2.2	0.0	95.6	0.0
shootable	0.9	0.9	0.0	2.8	2.8	0.9	70.1	21.5

Test set 3	null	clear	heavy-ppt	light-ppt	phase-sep	crystalline	optimisable	shootable
null	100.0	0.0	0.0	0.0	0.0	0.0	0.0	0.0
clear	2.4	97.4	0.0	0.0	0.0	0.2	0.0	0.0
heavy-ppt	1.7	2.5	64.2	25.8	0.3	0.0	5.5	0.0
light-ppt	3.8	4.2	3.4	86.0	1.3	0.8	1.3	0.0
phase-sep	11.3	4.4	0.0	9.6	54.7	1.1	19.0	0.0
crystalline	4.4	33.0	0.0	24.2	1.1	24.2	13.2	0.0
optimisable	0.0	25.9	0.0	0.0	0.0	3.7	70.4	0.0
shootable	50.0	5.6	0.0	0.0	0.0	16.7	27.8	0.0

Figure S10: Confusion matrix showing the results for each of the three test sets obtained using the ResNet50 classifier. Rows show class labels with predicted class in columns.

Test set 1	null	clear	heavy-ppt	light-ppt	phase-sep	crystalline	optimisable	shootable
null	87.8	4.4	3.1	3.4	0.0	0.3	0.3	0.7
clear	1.2	88.5	1.2	6.5	0.2	2.2	0.2	0.0
heavy-ppt	0.2	0.0	97.2	1.8	0.0	0.7	0.0	0.0
light-ppt	0.0	0.0	24.6	72.2	0.2	3.0	0.0	0.0
phase-sep	0.5	0.0	3.7	1.1	79.7	12.3	0.5	2.1
crystalline	0.3	0.5	0.5	0.3	0.0	96.8	0.0	1.6
optimisable	0.3	0.3	0.7	0.7	0.0	14.5	75.3	8.1
shootable	0.8	0.0	0.0	0.0	0.0	0.0	0.0	99.2

Test set 2	null	clear	heavy-ppt	light-ppt	phase-sep	crystalline	optimisable	shootable
null	72.1	1.9	8.7	11.8	0.0	3.7	0.6	1.2
clear	2.3	80.3	2.1	12.1	0.4	2.5	0.0	0.4
heavy-ppt	0.7	0.2	83.4	1.8	0.2	13.1	0.0	0.7
light-ppt	0.3	0.1	14.4	78.3	0.3	6.6	0.1	0.0
phase-sep	0.0	0.3	6.4	11.9	57.0	22.4	1.7	0.3
crystalline	0.0	0.4	3.4	3.8	0.0	90.6	0.4	1.5
optimisable	0.0	0.0	7.4	6.7	2.2	32.6	33.3	17.8
shootable	0.9	0.9	0.9	0.0	0.0	5.6	0.0	91.6

Test set 3	null	clear	heavy-ppt	light-ppt	phase-sep	crystalline	optimisable	shootable
null	25.0	8.3	8.3	50.0	0.0	0.0	8.3	0.0
clear	0.0	90.4	0.2	7.8	1.0	0.5	0.2	0.0
heavy-ppt	0.2	0.0	98.3	1.2	0.0	0.3	0.0	0.0
light-ppt	0.0	0.0	25.8	70.7	1.2	2.4	0.0	0.0
phase-sep	0.0	2.5	1.6	4.7	84.1	7.1	0.0	0.0
crystalline	0.0	2.2	1.1	1.1	0.0	95.6	0.0	0.0
optimisable	0.0	11.1	7.4	0.0	7.4	18.5	55.6	0.0
shootable	0.0	0.0	0.0	0.0	0.0	0.0	0.0	100.0

Figure S11: Confusion matrix showing the results for each of the three test sets obtained using the Xception classifier. Rows show class labels with predicted class in columns.

Test set 1	null	clear	heavy-ppt	light-ppt	phase-sep	crystalline	optimisable	shootable
null	94.6	0.3	2.0	2.0	0.3	0.3	0.0	0.3
clear	5.3	83.3	1.2	5.7	1.4	2.6	0.0	0.5
heavy-ppt	0.7	0.0	97.7	0.9	0.0	0.7	0.0	0.0
light-ppt	0.1	0.0	30.3	66.1	1.6	1.7	0.0	0.1
phase-sep	0.0	0.0	2.1	2.1	88.2	5.9	0.0	1.6
crystalline	0.0	0.3	1.3	2.4	2.4	91.3	1.1	1.3
optimisable	0.0	0.0	0.3	1.4	2.7	12.5	70.9	12.2
shootable	0.0	0.0	0.0	0.0	0.0	1.5	0.0	98.5

Test set 2	null	clear	heavy-ppt	light-ppt	phase-sep	crystalline	optimisable	shootable
null	73.3	0.0	11.8	14.3	0.0	0.6	0.0	0.0
clear	4.9	71.6	4.2	12.9	2.5	3.0	0.0	1.0
heavy-ppt	0.9	0.0	94.3	2.0	0.0	2.4	0.2	0.2
light-ppt	0.2	0.2	26.5	67.5	1.4	4.0	0.1	0.2
phase-sep	1.0	0.0	4.4	2.4	81.0	9.5	0.7	1.0
crystalline	0.0	0.0	22.5	7.1	1.1	62.9	2.3	4.1
optimisable	0.7	0.0	18.5	3.0	5.2	15.6	31.1	25.9
shootable	0.0	0.0	0.0	0.9	0.0	0.9	0.0	98.1

Test set 3	null	clear	heavy-ppt	light-ppt	phase-sep	crystalline	optimisable	shootable
null	25.0	0.0	50.0	16.7	8.3	0.0	0.0	0.0
clear	0.2	85.5	1.1	7.3	3.4	2.4	0.0	0.0
heavy-ppt	0.0	0.0	99.2	0.8	0.0	0.0	0.0	0.0
light-ppt	0.0	0.1	31.5	62.4	4.1	1.8	0.0	0.1
phase-sep	0.0	0.0	0.3	0.6	96.4	1.9	0.0	0.8
crystalline	0.0	0.0	0.0	4.4	5.5	89.0	1.1	0.0
optimisable	0.0	0.0	3.7	0.0	14.8	29.6	44.4	7.4
shootable	0.0	0.0	0.0	0.0	5.6	0.0	0.0	94.4

Figure S12: Confusion matrix showing the results for each of the three test sets obtained using the InceptionV3 classifier. Rows show class labels with predicted class in columns.
